# Dentin tubule orientation determines odontoblastic differentiation *in vitro*: A morphological study

**DOI:** 10.1371/journal.pone.0215780

**Published:** 2019-05-09

**Authors:** José Javier Martín-de-Llano, Manuel Mata, Santiago Peydró, Amando Peydró, Carmen Carda

**Affiliations:** 1 Department of Pathology. Faculty of Medicine and Odontology, University of Valencia, Valencia, Spain; 2 Fundación para la Investigación del Hospital Clínico de la Comunidad Valenciana (INCLIVA), Valencia, Spain; 3 Centro de Investigación Biomédica en Red de Enfermedades Respiratorias (CIBERES), Madrid, Spain; 4 Centro de Investigación Biomédica en Red en Bioingeniería, Biomateriales y Nanomedicina (CIBERBBN), Madrid, Spain; Università degli Studi della Campania, ITALY

## Abstract

Odontoblasts are post-mitotic cells responsible for maintenance of the dentin, and are therefore important for dental health. In some cases, irreversible pulpitis leads to necrosis and consequently death of odontoblasts. Regenerative endodontics (RE) uses the concept of tissue engineering to restore the root canals to a healthy state, allowing for continued development of the root and surrounding tissue. Human dental pulp stem cells (hDPSCs) have been successfully used in RE to restore odontoblast function. Surface microgeometry is one of the most important factors involved in the induction of differentiation of hDPSCs into odontoblast-like cells. Although different authors have demonstrated the importance of a dentin-like surface with accessible dentin tubules to induce differentiation of hDPSCs, the ultrastructural characteristics of the cells and the secreted extracellular matrix have not been studied in depth. Here, we used an acellular dentin scaffold containing dentin tubules in different spatial geometries, which regulated their accessibility to cells. hDPSCs were cultured on the scaffolds for up to 6 weeks. Systematic characterization of differentiated cells was performed using both optical (hematoxylin and eosin, Masson trichrome, and immunohistochemical determination of dentin sialoprotein [DSSP]) and transmission electron microscopy. The results presented here indicated that cells grown on the dentin surface containing accessible dentin tubules developed a characteristic odontoblastic phenotype, with cellular processes similar to native odontoblasts. The cell organization and characteristics of secreted extracellular matrix were also similar to those of native dentin tissue. Cells grown on non-accessible dentin tubule surfaces secreted a more abundant and dense extracellular matrix, and developed a different phenotype consisting of secretory flat cells organized in layers. Cells grown far from the scaffold, i.e., directly on the culture well surface, developed a secretory phenotype probably influenced by biochemical factors released by the dentin scaffold or differentiated cells. The results presented here support the use of hDPSCs to regenerate dentin and show the utility of scaffold microgeometry for determining the differentiation and secretory phenotype of cultured cells.

## Introduction

The dental pulp is a specialized connective tissue containing the cells that synthesize dentin, the odontoblasts, in addition to the typical elements of loose connective tissue. In vital teeth, these cells continue depositing dentin matrix during the lifetime of the individual [1].

Inflammation of the dental pulp (pulpitis) may be caused by different pathologies, including caries, periodontal disease, and trauma [2–3]. If the pathological stimulus is untreated, the pulpitis can be irreversible, leading to pain, tissue necrosis, and abscess formation [4]. If the antiinflammatory mechanisms of the pulp tissue fail, necrosis occurs and odontoblasts die [5]. In some cases, mesenchymal cells present in the pulp can differentiate into odontoblast-like cells and secrete reparative dentin, which is quite different from reactionary dentin. It may contain cellular inclusions, which resemble the osteocytes found in bone tissue. In addition, its extracellular matrix contains some non-collagenous proteins, such as osteopontin, which are more typical of bone than dentin [6].

Root canal therapy (RCT) is an alternative clinical approach to treat this pathology [7]. Conventional RCT involves extirpation of injured pulp, removal of cell debris from the root using different compounds, including chlorhexidine gluconate, and filling the root canal with a material, usually gutta-percha [8]. RCT is a valuable technique that can save teeth that would otherwise be condemned to extraction due to different causes, but it is not without disadvantages. Previous studies have demonstrated that the presence or absence of dental pulp is a key factor affecting the prognosis of affected teeth. The hazard ratio for tooth loss increases by 7.4-fold for pulpless molars and by 1.8-fold for pulpless anterior teeth or premolars [9–11]. Moreover, endodontically treated teeth are susceptible to reinfection and fractures caused by the loss of significant amounts of dentin, especially when clinicians are dealing with dental trauma or an open apex tooth, as demonstrated in clinical trials [12–13].

These disadvantages have driven the development of novel alternative treatments centered around regeneration of the dentin–pulp complex, known as regenerative endodontics (RE). The RE concept was developed more than 50 years ago and was initially associated with revascularization procedures. Nevertheless, the intense research activity in the field of pulp regeneration has enabled translation of new therapeutic methodologies to the clinic [14–15].

Different stem cells have been used for regeneration of the dentin–pulp complex, including those derived from dental pulp tissue (human dental pulp stem cells, hDPSCs), exfoliated deciduous teeth (stem cells from human exfoliated deciduous teeth, SHEDs), periodontal ligament (periodontal ligament stem cells, PDLSCs), and stem cells from the apical papilla, SCAPs) [16].

These are self-renewing mesenchymal stem cells (MSCs) some of them residing within the perivascular niche of the dental pulp [17–19], and are thought to originate from the cranial neural crest and express both MSC and neural stem cell markers [20]. Neural crest cells originate during the formation of neural tube, at the 3^rd^ week of embryo development, then undergo an epithelial to mesenchymal transition (EMT) and migrate to different body compartments generating the majority of craniofacial tissues [21–23]. hDPSCs can be obtained easily from extracted third molars, and under specific conditions they can differentiate into a variety of cell types different from odontoblasts, including neurons, osteoblasts, adipocytes, chondrocytes or angiogenic linages among others [24–28]. Their high proliferation rate and proven odontoblastic potential make hDPSCs suitable for use in RE [16, 22, 29–32]. Nevertheless, it should be noted that this odontoblastic potential is dependent on different factors, including the scaffold microstructure and composition of the culture medium. With regard to scaffold microstructure, some authors have reported that a highly porous structure induces cell attachment, proliferation, and differentiation into odontoblast-like phenotypes, as reported previously using collagen sponges, poly-l-lactic acid, and chitosan, among other materials [33–35]. The presence of a scaffold mimicking the geometry of the natural dentin also determines the differentiation of hDPSCs; that is, hDPSCs in contact with acellular dentin developed monopolar cytoplasmic processes within dentin tubules when they were accessible to cells [36–37]. Although these authors described the differentiation of hDPSCs to odontoblast-like cells using optical microscopy methods, no fine ultrastructural studies have been performed and further studies are needed to characterize the odontoblast-like cells differentiated from hDPSCs.

In this study, we used decellularized dentin-based scaffolds containing dentin tubules in different spatial geometries regulating the accessibility to cells. hDPSCs have been cultured on different surfaces of dentin scaffolds for up to 6 weeks. Systematic characterization of differentiated cells was performed using both optical (hematoxylin and eosin and Masson trichrome staining, and immunohistochemical determination of dentin sialoprotein [DSSP]) and transmission electron microscopy (TEM). We found substantial differences in the ultrastructural and secretory phenotypes of differentiated cells depending on the orientation of dentinal tubules of dentin scaffolds, and subsequently in the accessibility of dentin tubules to cultured cells. The results presented here support the use of hDPSCs to regenerate dentin and demonstrate the effects of scaffold microgeometry in determining the differentiation and secretory phenotype of hDPSCs.

## Methods

### Cell culture

hDPSCs were isolated as described previously [26]. All donors provided informed consent. The study was conducted in accordance with the Declaration of Helsinki and applicable local regulatory requirements and laws. All procedures were approved by the Ethics Committee of the University of Valencia (Spain). The dental pulp of human third molars was gently removed under aseptic conditions using cow horn forceps with a small excavator, and immersed in culture tubes filled with culture medium. The specimens were then divided into small pieces using a bistoury blade, immersed in HBSS, and incubated for 2 hours at 37°C in an atmosphere of 5% CO_2_ and 95% air. The supernatant was removed, and 0.1% type I collagenase and dispase (Sigma-Aldrich, Madrid, Spain) was added for 15 minutes, followed by centrifugation at 1,500 rpm for 10 minutes. The supernatant was removed, and the cells were plated in 25-cm^2^ flasks in αMEM culture medium (Sigma-Aldrich) containing penicillin/streptomycin, 10% FCS (Sigma-Aldrich), amphotericin B, 2 mM l-glutamine, and 100 μM ascorbic acid (Sigma-Aldrich). The medium was replaced every 3–4 days. Once the cells reached confluence, flow cytometry was performed.

### Flow cytometric characterization of hDPSCs

hDPSCs were characterized using a cytometer equipped with a 488-nm Argon laser and a 635-nm red diode laser, as described previously [38]. Experimental data were analyzed using CellQuest software (Becton Dickinson, Madrid, Spain). To exclude debris, samples were gated based on their light-scattering properties in the side- and forward-scattered light modes, and 10,000 events per sample within this gate (R1) were recorded using the medium setting for the sample flow rate. The following markers were evaluated: CD29 (Alexa Fluor 488), CD31 (PE/Cy7), CD44 (PE/Cy5), CD45 (Pacific Blue), CD105 (APC), and CD146 (PE). Almost 98% of the cells analyzed were positive for CD29, CD44, CD105, and CD146 but negative for CD31 and CD45 (S1 Fig).

### Scaffold preparation and study design

Dentin scaffolds were generated using extracted teeth. Non-endodontic teeth were used in this study. All of the specimens used had an intact pulpal cavity, no caries, and showed good preservation of the crown and apex. Six incisors, two premolars, and seven molars were used. All donors provided informed consent. The study was conducted in accordance with the Declaration of Helsinki and applicable local regulatory requirements and laws. All procedures were approved by the Ethics Committee of the University of Valencia (Spain). Following extraction, specimens were stored at 4°C in Dulbecco’s phosphate buffered saline (DPBS) supplemented with a solution consisting of 10% antibiotics and fungizone. Teeth were then washed with chlorhexidine and an ultrasonication apparatus (Cavitron; Satelec, Cedex, France), and a 7/8 Gracey curette was used to remove the periodontal ligament, dental calculus and bacterial biofilm.

Cross-sectioning to the cementoenamel junction was performed using a diamond disk to separate the dental crown and root. The apex was then removed and a diamond bur was used to obtain access to the pulp camber. Pulp tissue was removed using barbed broaches. To completely remove the pulp tissue and predentin covering the pulp chamber, a process similar to that used for root canal treatment was used. Briefly, endodontic filers of different diameters were used to clean and expand the root canal. Then, 0.12% chlorhexidine solution was used to clean the dentin surface.

**Fig 1:**
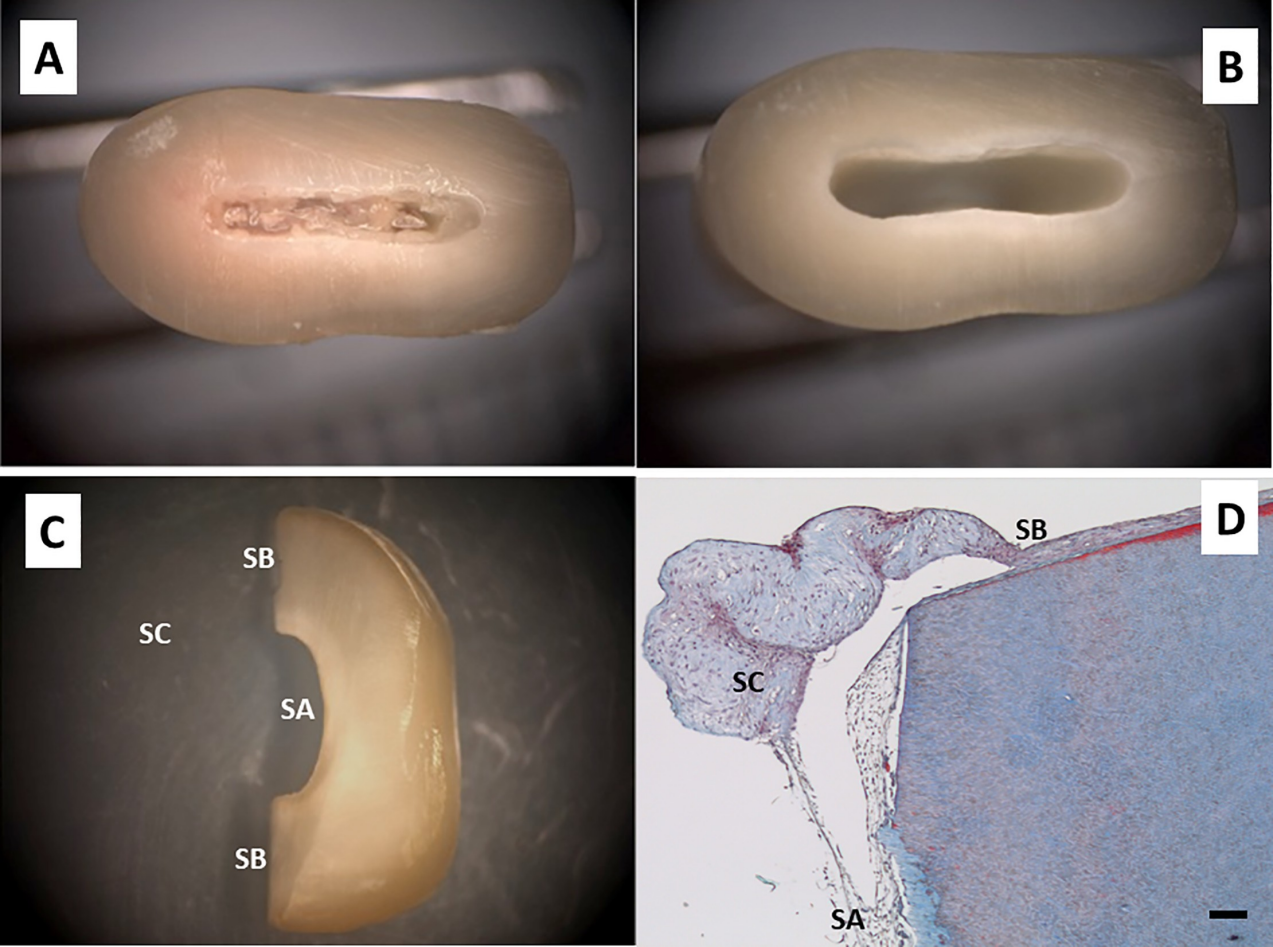
Dentin acellular scaffold preparation. Extracted specimens were washed with chlorhexidine solution and periodontal bacterial biofilm and dental calculus were removed. Cross-sectioning to the cementoenamel junction was performed and the apex was removed (panel A). Pulp tissue and predentin were completely removed (panel B). Sections of teeth 1.5-mm thick were obtained and cut into two to three fragments (panel C). Three different surfaces were considered: surface A (SA: dentin tubules perpendicular or oblique to the surface of the scaffold, i.e., accessible tubules), surface B (SB: dentin tubules parallel to the surface of the scaffold, i.e., non-accessible tubules), and surface C (SC: cells grown far from the scaffold, i.e., cells grown on the culture plate surface). Human dental pulp stem cells (hDPSCs) were cultured on the scaffolds for up to 6 weeks and processed for light microscopy. Samples were embedded in paraffin, cut into sections, and subjected to Masson’s trichrome staining (panel D). Representative results of 15 different specimens are shown. Scale bar equals to 100 μm.

A diamond disk was used to obtain 1.5-mm thick transversal sections of the roots of processed teeth, which were cut into two to three fragments (Fig 1, panels A–C). Specimens were washed for 1 hour at room temperature (RT) with chlorhexidine solution, and then washed extensively five times with DPBS supplemented with antibiotics and an antifungal agent at 4°C for up to 72 hours.

hDPSCs were cultured on scaffolds with different dentin tubule orientations according to the surface type. 5.000 cells resuspended in 1.5 ml of culture medium were seeded and cultured on the scaffolds for up to 6 weeks. Every 2 weeks, a group of samples were processed for optical and electron microscopy. The morphology and secretory activity of cultured cells were compared according to the orientation of the dentin tubules with regard to the surface of the scaffold. Cells grown out of the scaffold were also examined. Three different experimental groups were considered according to the scaffold surface, as shown in Fig 1C. Cells grown on surface A (SA: dentin tubules perpendicular or oblique to the surface of the scaffold, i.e., accessible tubules), cells grown on surface B (SB: dentin tubules parallel to the surface of the scaffold, i.e., non-accessible tubules), and cells grown on surface C (SC: cells grown far from the scaffold, i.e., cells grown on the culture plate surface). A panoramic section representative of these surfaces with cultured cells is shown in Fig 1D. All experiments were performed using six replicates with hDPSCs isolated from two different donors.

### Light microscopy: Determination of dentin sialoprotein expression

Specimens were washed with DPBS and fixed in 4% buffered formaldehyde solution for 3 hours at 4°C. Decalcification of scaffolds was performed by incubation in a solution containing 90% Osteosoft (Merck KGaA, Darmstadt, Germany) and 10% of the above formaldehyde solution for up to 21 days. Samples were then embedded in paraffin, cut into sections, and subjected to hematoxylin and eosin and Masson’s trichrome staining according to standard protocols.

DSSP expression was evaluated by immunohistochemistry using a mouse anti-human antibody (LFMb-21, dilution 1:50; Santa Cruz Biotechnology, Santa Cruz, CA) according to the manufacturer’s instructions. Sections were deparaffinized and rehydrated through a graded ethanol series, rinsed in distilled water, and treated with 0.3% H_2_O_2_ and 10% normal horse serum to block endogenous peroxidase and nonspecific binding, respectively. The Envision amplification system (Envision System + labeled polymer-HRP anti-mouse; Dako, Carpinteria, CA, USA) was used, followed by development with 3,3′-diaminobenzidine (Dako) as a chromogen according to the manufacturer’s instructions, which yielded brown staining in immunoreactive structures. Sections were finally counterstained with Mayer’s hematoxylin (Sigma-Aldrich).

### Transmission electron microscopy

hDPSCs were cultured on dentin scaffolds, washed with DPBS, and fixed in 2.5% glutaraldehyde solution for 4 hours at 4°C. The samples were then decalcified in 4% EDTA for up to 21 days and washed with a solution containing 9% Millonig buffer, 9% calcium chloride, and 1% glucose (Sigma-Aldrich). After fixation in 2% osmium tetroxide solution, the samples were included in Epon 812 (TAAB Laboratories Equipment Ltd., Aldermaston, UK) and processed for electron microscopy, as reported previously [39]. A JEM 1010 electron microscope (JEOL, Tokyo, Japan) operated at 60 kV was used to obtain ultrastructural images.

## Results

### Optimization of the experimental model

**Fig 2:**
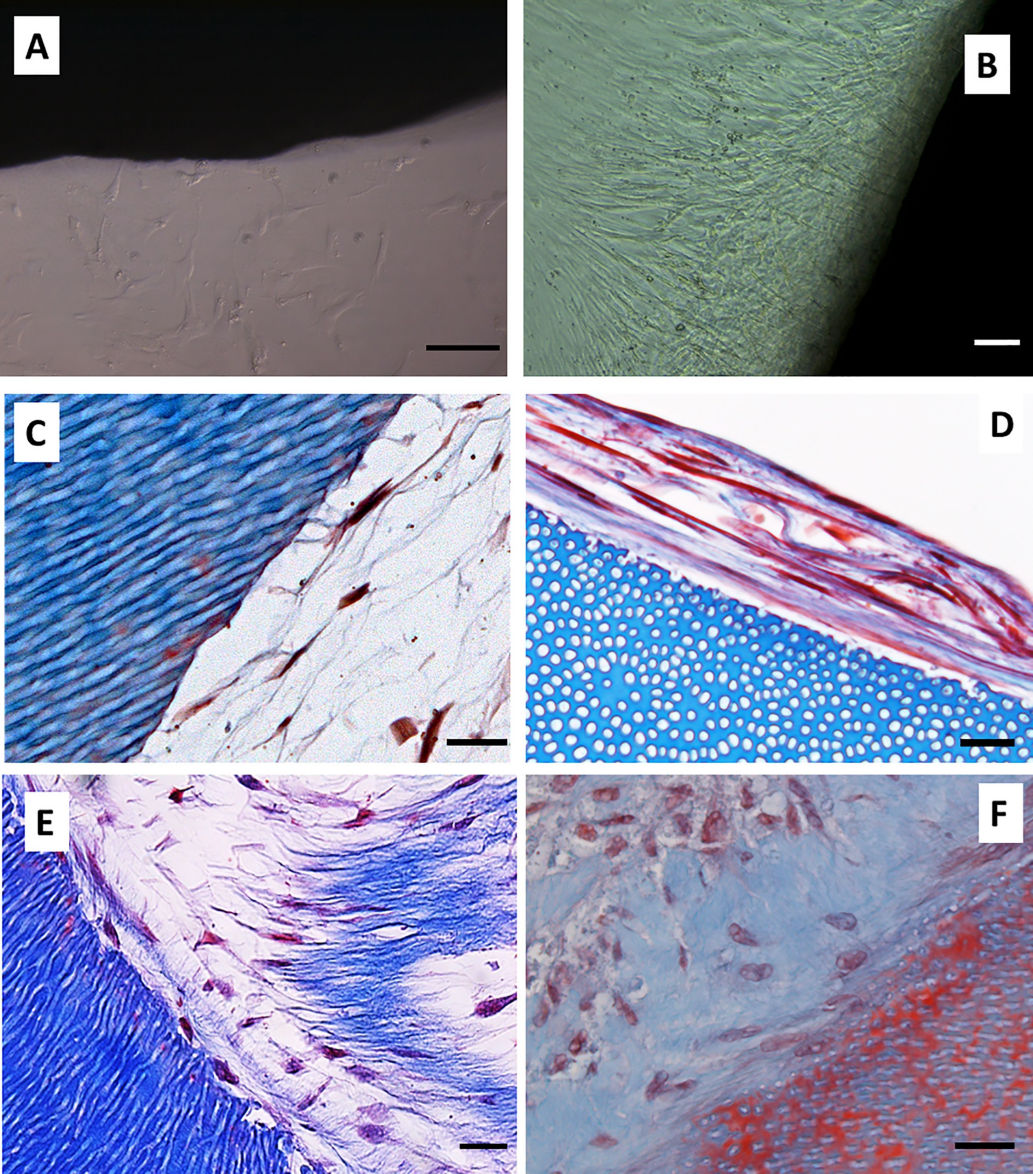
Cell culture kinetics on acellular dentin scaffolds. hDPSCs were cultured on the scaffolds for up to 6 weeks and processed for light microscopy. Confluence was estimated by phase contrast microscopy. Representative images obtained at 1 week (panel A) and 6 weeks (panel B) are shown. Samples were embedded in paraffin, cut into sections, and subjected to Masson’s trichrome staining. Representative images obtained at 2 weeks (panels C and D) and 6 weeks (panels E and F) of cells grown on SA (panels C and E) and SB (panels D and F) are shown. All experiments were performed in six replicates using hDPSCs isolated from two different donors. Scale bar equals to 20 μm.

After preparation according to method described, the scaffolds were placed in 24-well culture plates and 2,500 hDPSCs/cm^2^ was added in 1.5 ml of culture medium. This volume was sufficient to completely cover the dentin scaffold. The cells were then cultured for up to 6 weeks. Confluence was estimated every 2 weeks by phase contrast microscopy. All of the samples reached confluence 6 weeks after seeding. Representative images are shown in Fig 2A (1 week) and 2B (6 weeks).

Cell morphology was then evaluated by light microscopy. Samples from week 1 could not be examined by microscopy because of the poor cell adhesion to the surface of the dentin. Two-week samples were processed and serial sections were obtained. Cells grown on the SA became stellate and generated a reticular extracellular matrix (Fig 2C). Conversely, hDPSCs grown on SB showed a flat morphology forming layers of cohesive cells (Fig 2D). Although the new tissue formed on the surface of the scaffolds from week 2 to 6 increased in thickness, no substantial changes in cell morphology were evident under optical microscopy (Fig 2E and 2F).

Based on the proliferation and light microscopy results, the 6-week experimental time point was chosen for the rest of the experiments.

### Cellular morphology and extracellular matrix characteristics of hDPSCs grown on surface A

**Fig 3:**
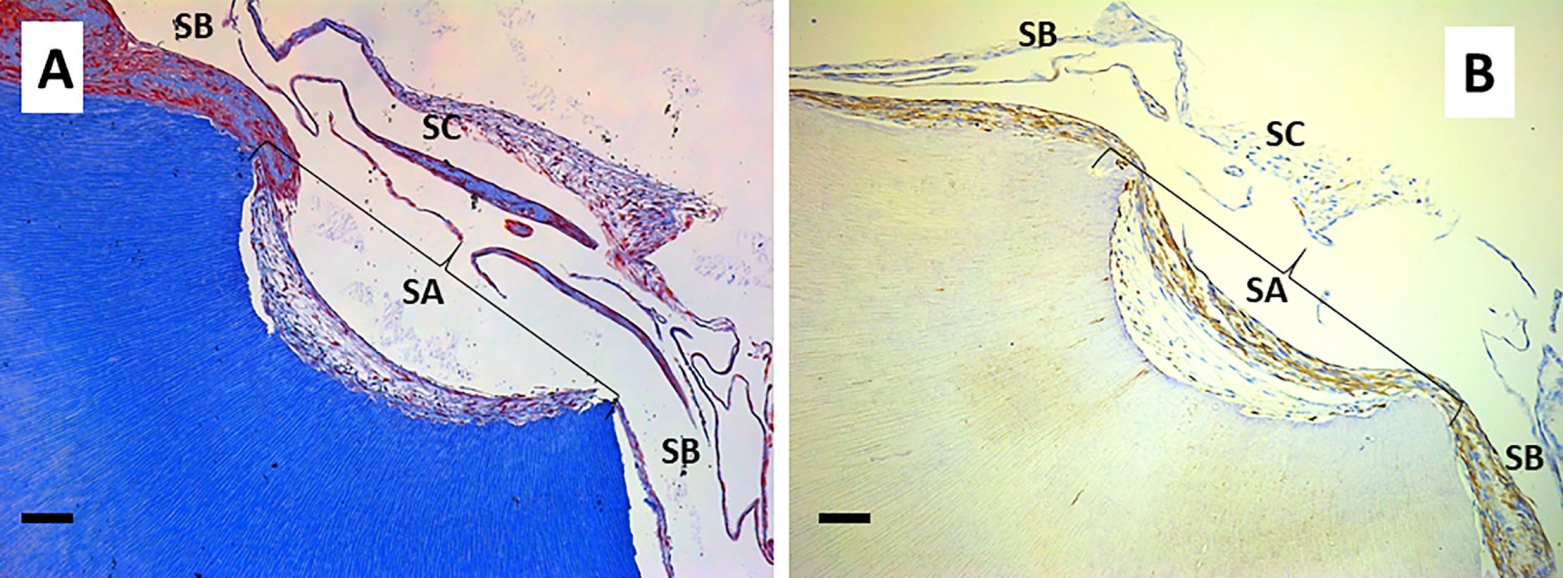
Extracellular matrix secreted by hDPSCs cultured on SA. hDPSCs were cultured on scaffolds for 6 weeks and processed for light microscopy. Samples were embedded in paraffin, cut into sections, and subjected to Masson’s trichrome staining (panel A) or immunohistochemistry for dentin sialophosphoprotein (DSPP) (panel B). The area between SA (center) and SB (left and right) is shown. Representative results of 12 separate experiments are shown. Scale bar equals to 100 μm.

Next, the morphology of cells grown on the surfaces of dentin scaffolds containing accessible (perpendicular or oblique) dentin tubules was evaluated. Stellate cells surrounded by a reticular extracellular matrix containing thin collagen fibers were found on these surfaces (Fig 3A); this image shows the area between SA (center) and SB (left and right). It is important to highlight the different compositions of the extracellular matrix secreted by cells. Immunohistochemical analysis of dentin sialophosphoprotein (DSPP) revealed deposits of this protein in both surfaces (Fig 3B). Nevertheless, the deposits found in the reticular matrix associated with SA were smaller than those found on the SB surface.

**Fig 4:**
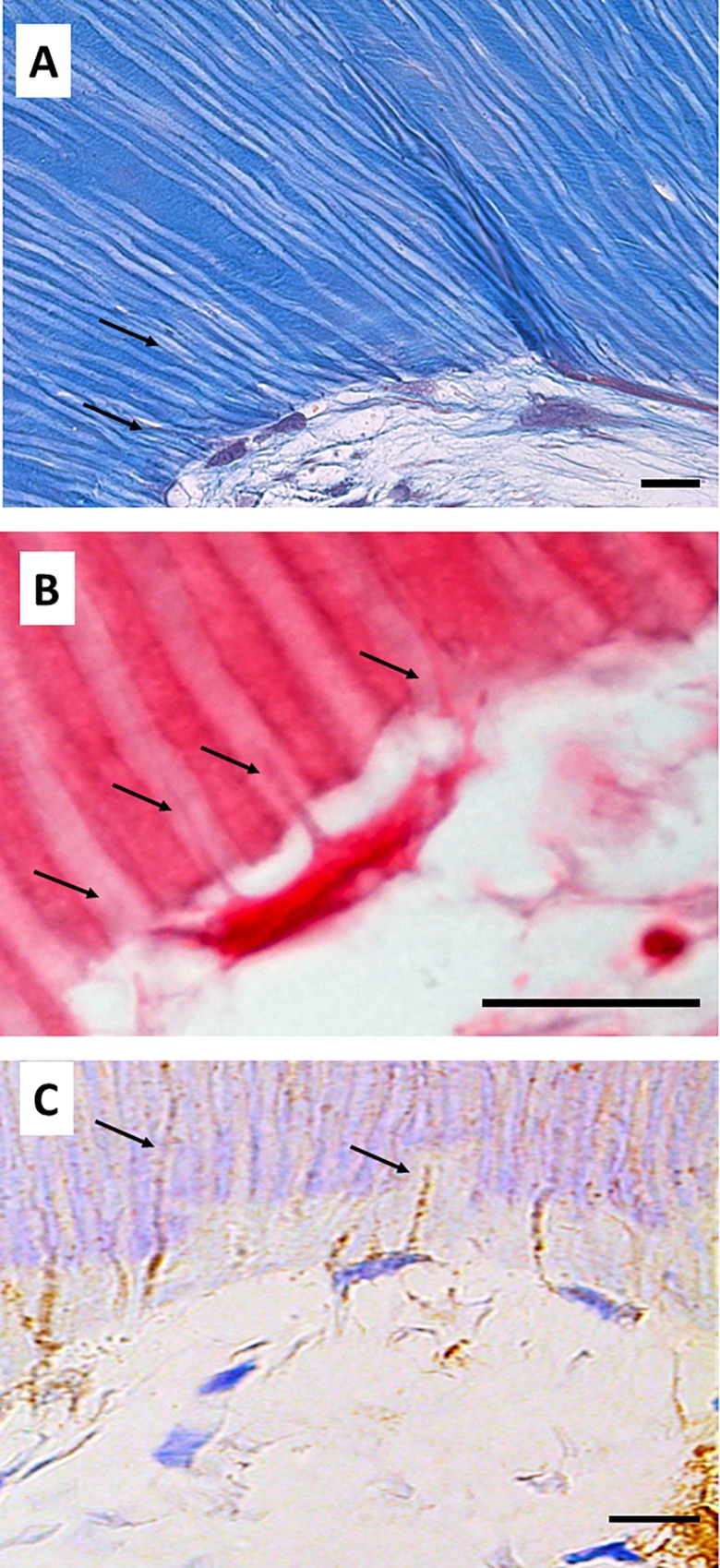
Cell morphology of hDPSCs cultured on SA. hDPSCs were cultured on scaffolds for 6 weeks and processed for light microscopy. Samples were embedded in paraffin, cut into sections, and subjected to Masson’s trichrome (panel A), hematoxylin and eosin staining (panel B), or immunohistochemistry for DSPP (panel C). Black arrows indicate cellular processes inside dentin tubules. Representative results of 12 separate experiments are shown. Scale bar equals to 20 μm.

With regard to cell morphology, odontoblast-like cells were found in contact with the SA surface. These cells showed a flat morphology and grew perpendicular to the dentin scaffold surface. Several cellular processes were observed inside dentin tubules (Fig 4A). Interestingly, some of the cells generated more than one process, as shown in Fig 4B. These odontoblast-like cells were positive for DSPP, which was detected in the cell processes occupying the dentin tubules (Fig 4C).

**Fig 5:**
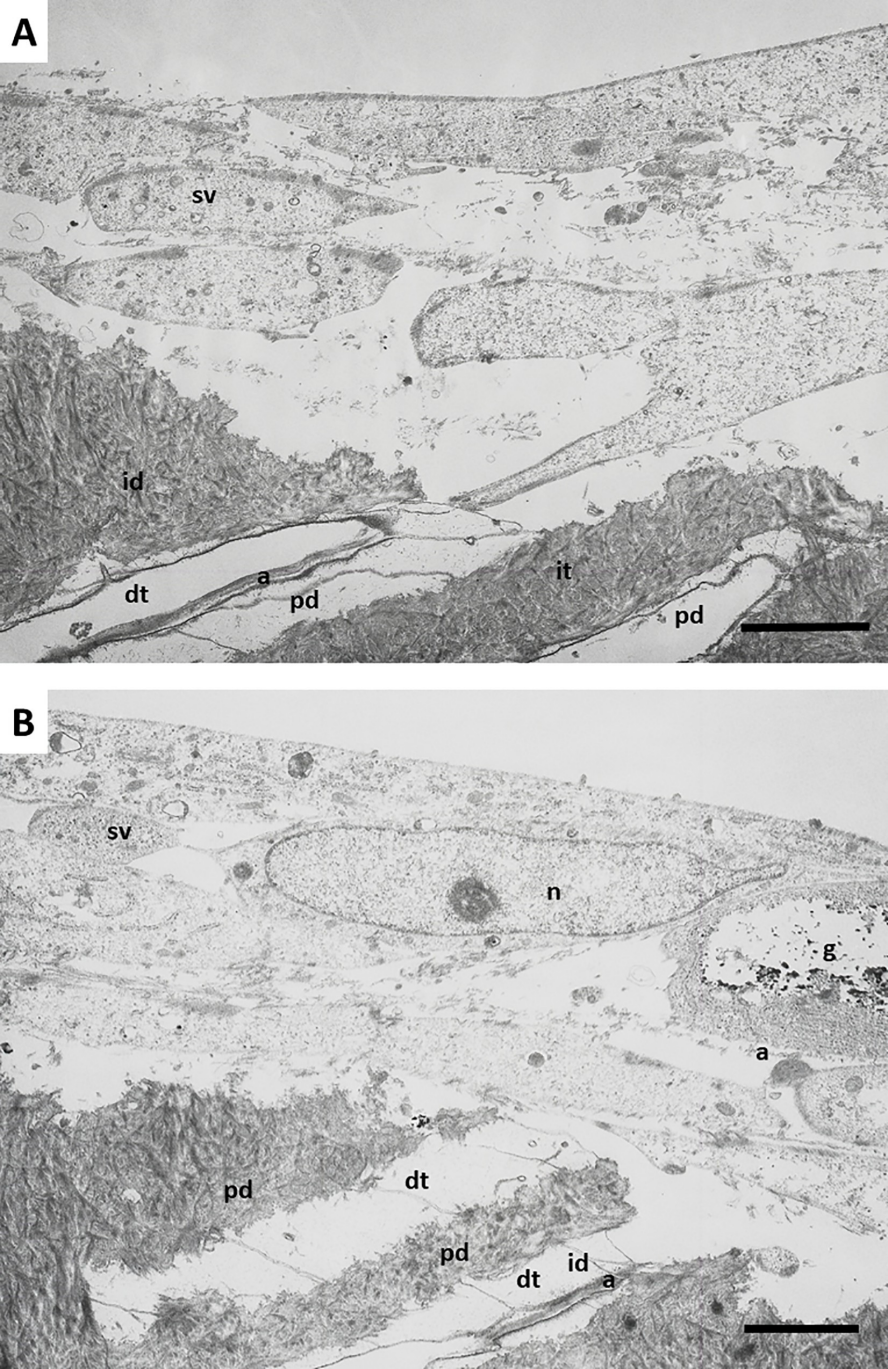
Ultrastructure of hDPSCs cultured on SA. hDPSCs were cultured on scaffolds for up to 6 weeks and processed for transmission electron microscopy (TEM). dt, dentin tubule; id, intertubular dentin; pd, peritubular dentin; cp, cellular process; a, actin filaments; g, glycogen; n, nucleus; sv, secretory vesicles. Representative results of 12 separate experiments are shown in panel A and B. Scale bar equals to 5 μm.

TEM analysis confirmed and extended these findings. Odontoblast-like cells extending processes inside the dentin tubules were found on the surface of the dentin scaffolds. Large amounts of actin filaments were observed inside these neo-processes, which may have been participating in anchorage of the cells to the dentin matrix (Fig 5). Differences in the composition of dentin were also observed. As shown in Fig 5, the intertubular dentin was more electron-dense and contained more collagen fibers than peritubular dentin, which was less dense and contained smaller amounts of collagen fibers.

**Fig 6:**
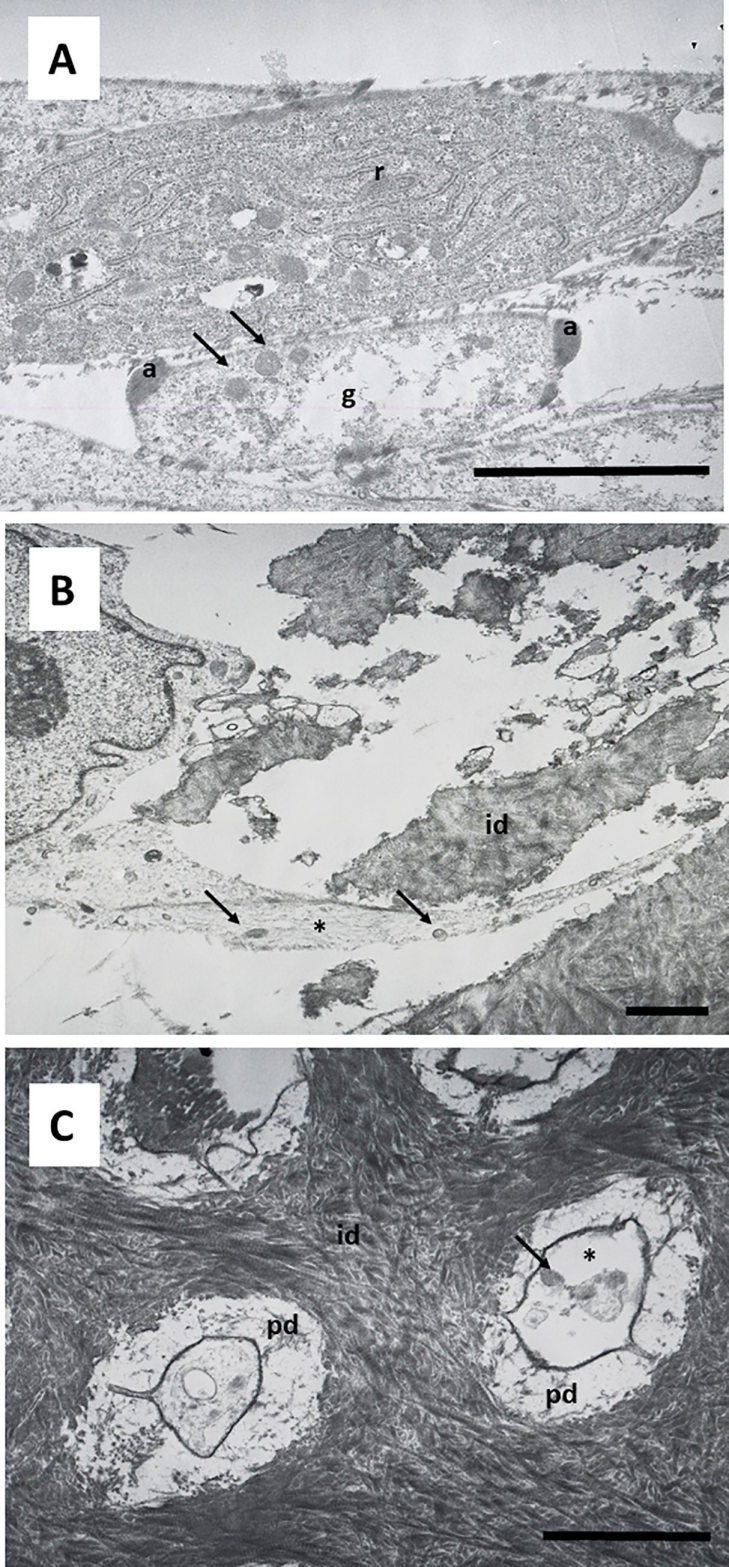
Ultrastructure of hDPSCs cultured on SA. hDPSCs were cultured on scaffolds for up to 6 weeks and processed for TEM. r, rough endoplasmic reticulum; a, actin filaments; g, glycogen; id, intertubular dentin; pd, peritubular dentin; dt, dentin tubule; sv, secretory vesicles; cp, cellular process. Representative results of 12 separate experiments are shown in panel A, B and C. Scale bar equals to 5 μm.

**Fig 7:**
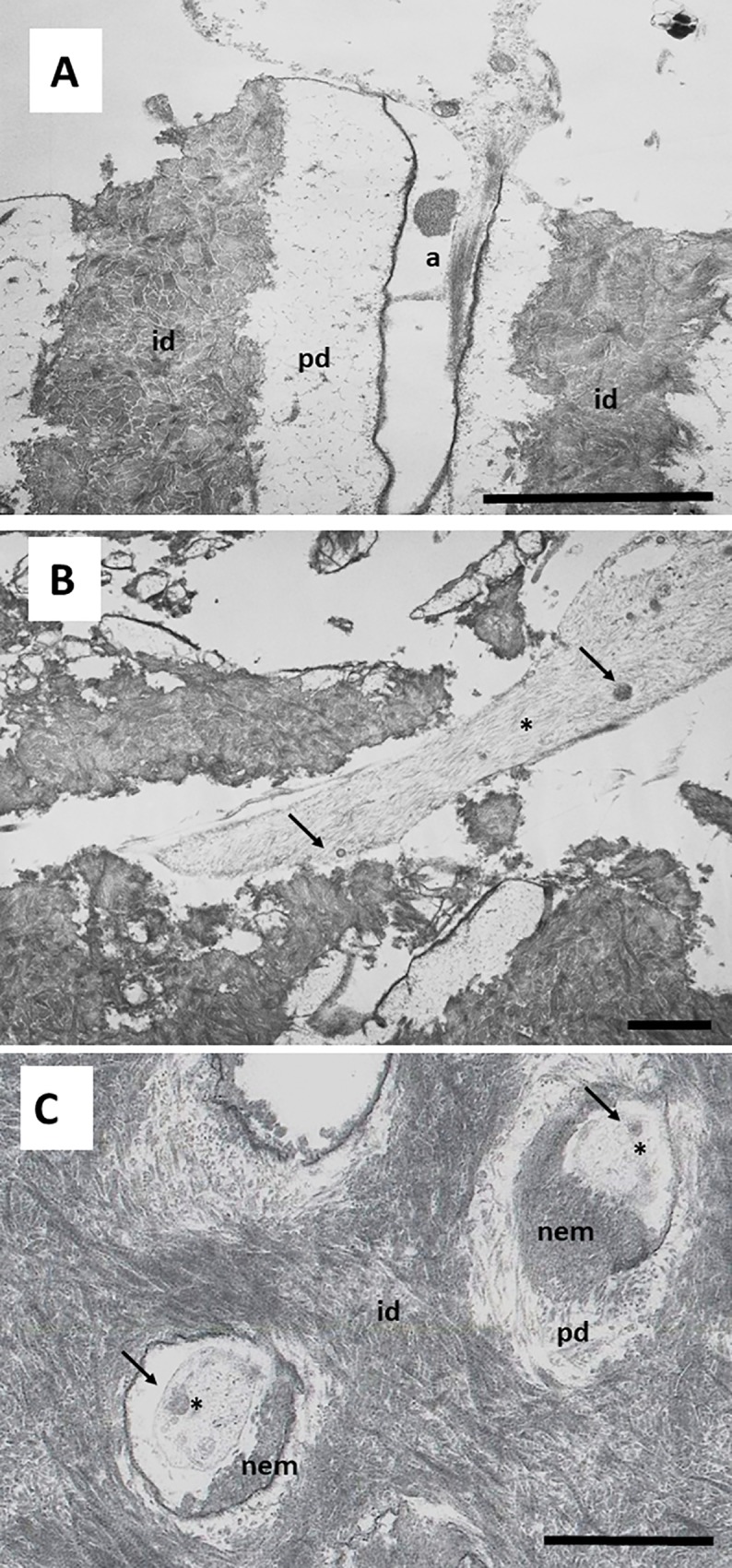
Ultrastructure of hDPSCs cultured on SA. hDPSCs were cultured on scaffolds for 6 weeks and processed for TEM. r, rough endoplasmic reticulum a, actin filaments; g, glycogen; id, intertubular dentin; pd, peritubular dentin; dt, dentin tubule; sv, secretory vesicles; cp, cellular process; nem, neo-secreted extracellular matrix. Representative results of 12 separate experiments are shown in panel A, B and C. Scale bar equals to 5 μm.

**Fig 8:**
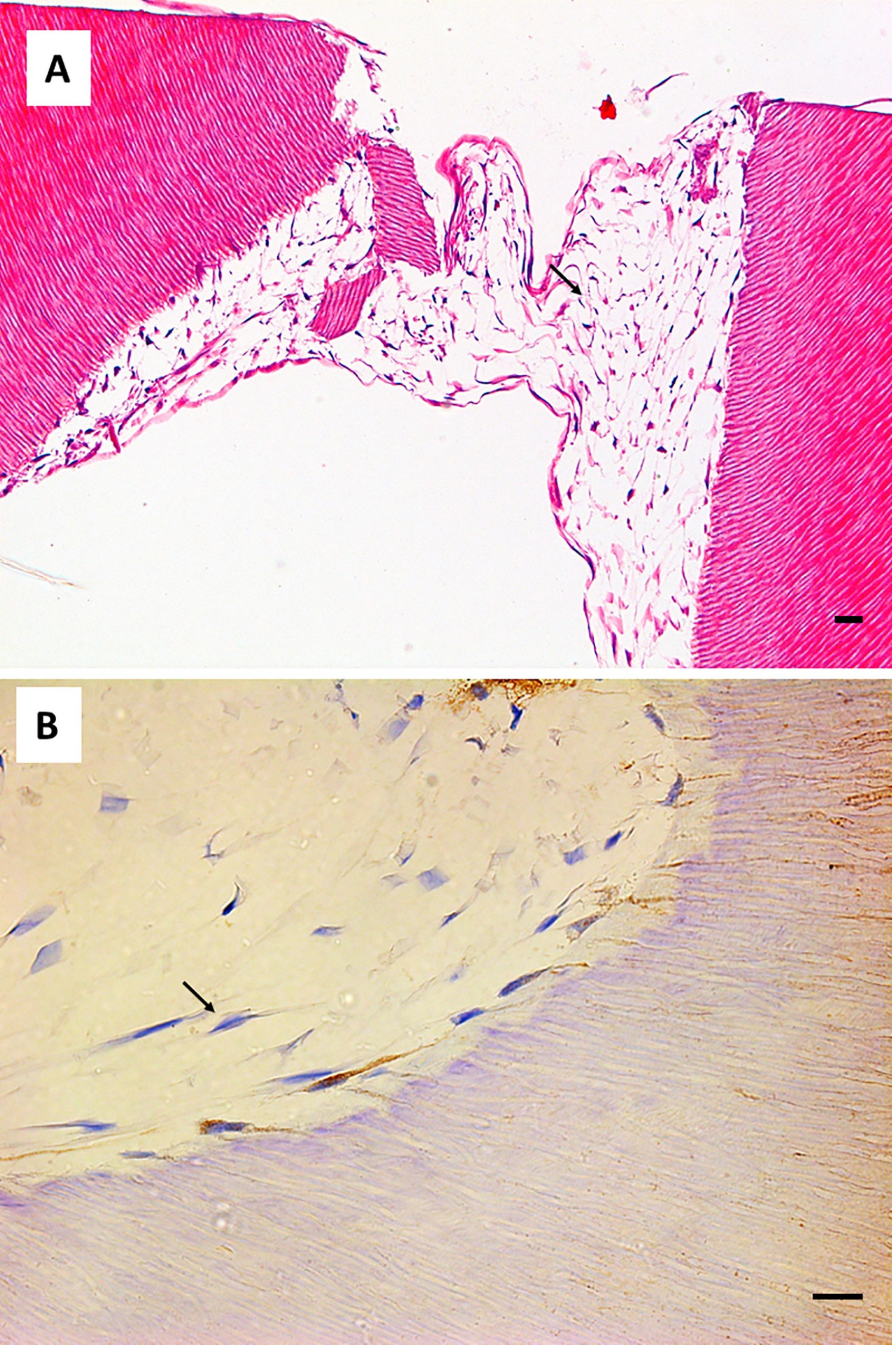
Cell morphology of hDPSCs cultured on SA. hDPSCs were cultured on scaffolds for 6 weeks and processed for light microscopy. Samples were embedded in paraffin, cut into sections, and subjected to hematoxylin and eosin staining (panel A) or immunohistochemistry for DSPP (panel B). Black arrows indicate stellate cells negative for DSPP. Representative results of 12 separate experiments are shown. Scale bar equals to 20 μm.

Cells grown on this surface showed a characteristic secretory phenotype, with a large and irregular cytoplasm, non-condensed chromatin, large nucleus, evident nucleolus, spherical and electron-dense secretory vesicles, and accumulation of glycogen (Fig 5B). Dense bundles of actin filaments were observed in the areas of anchorage to the dentin surface, and at the sites of intercellular junctions (Fig 5B). A well-developed rough endoplasmic reticulum (RER) was also characteristic of these cells (Fig 6A). Detailed analysis revealed that secretory vesicles were present not only in the perinuclear cytoplasm, but also in the cell processes inside dentin tubules, as shown in Fig 6B. In some preparations, active secretion of extracellular components was also identified, as shown in Fig 6C. A more detailed analysis of the cellular processes inside the dentin tubules revealed a conical shape with bundles of actin filaments and a well-developed cytoskeleton. These processes almost filled the dentin tubules (Fig 7A and 7B). This developed cytoskeleton and the presence of secretory vesicles were also observed in analysis of cross-sections of dentin tubules containing cell processes. In some tubules, different stages of the secretory process of extracellular matrix generation on the dentin tubule wall were observed (Fig 7C). Together with these odontoblast-like cells, stellate cells were found inside the reticular matrix. These cells were negative for DSPP (Fig 8A and 8B).

### Cellular morphology and extracellular matrix characteristics of hDPSCs grown on surface B

**Fig 9:**
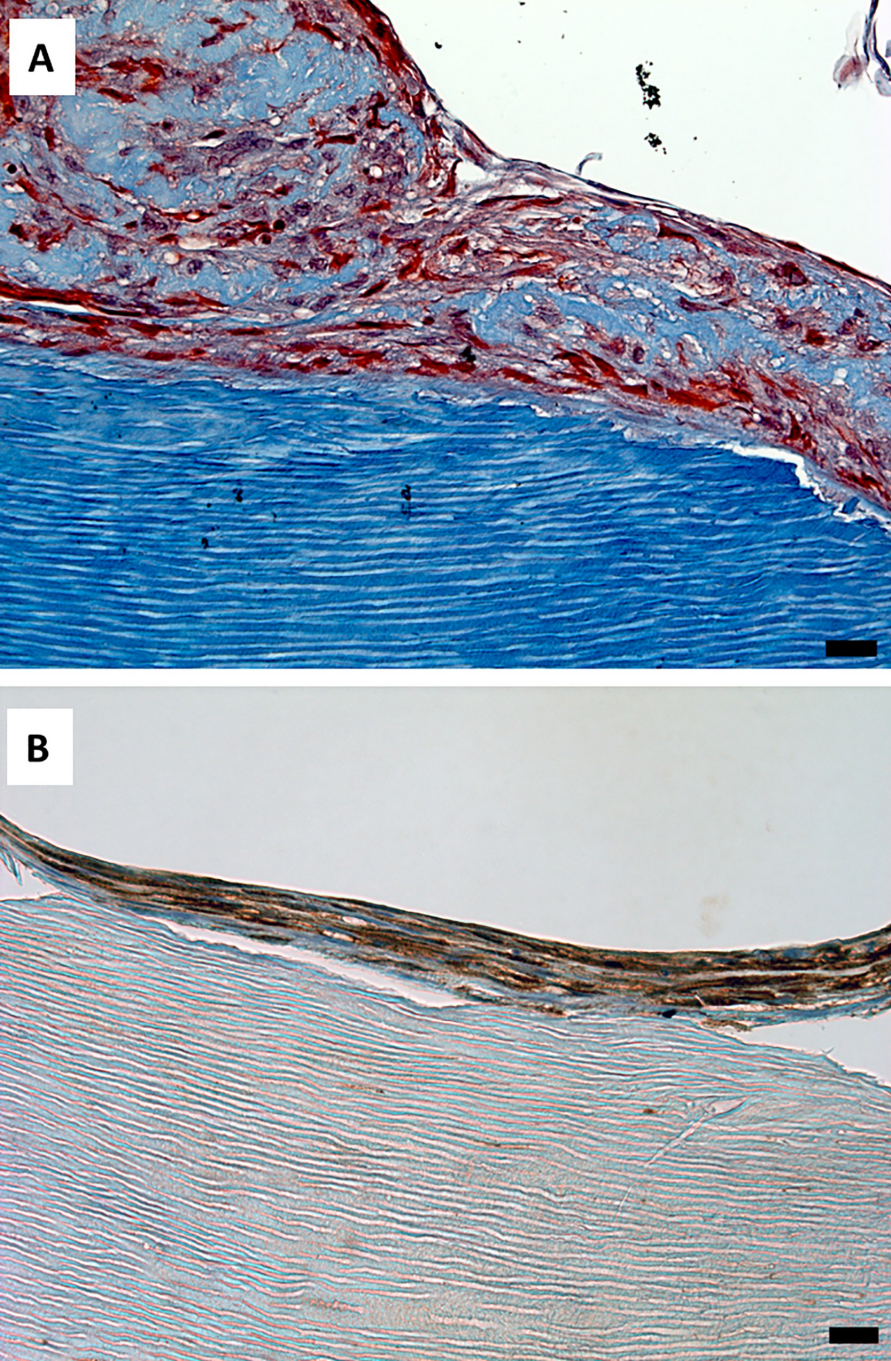
Cell morphology of hDPSCs cultured on SB. hDPSCs were cultured on scaffolds for 6 weeks and processed for light microscopy. Samples were embedded in paraffin, cut into sections, and subjected to Masson’s trichrome staining (panel A) or immunohistochemistry for DSPP (panel B). Representative results of 12 separate experiments are shown. Scale bar equals to 20 μm.

The morphology of the hDPSCs grown on dentin scaffolds containing non-accessible (parallel) dentin tubules was also examined. These flat cells arranged generating several highly cohesive layers of cells and extracellular matrix. This dense extracellular matrix had a higher collagen content than that observed on surfaces with dentin tubules accessible to cells (Fig 9A). Positive staining for DSPP was also found both inside the cells and forming deposits in the extracellular matrix (Fig 9B).

**Fig 10:**
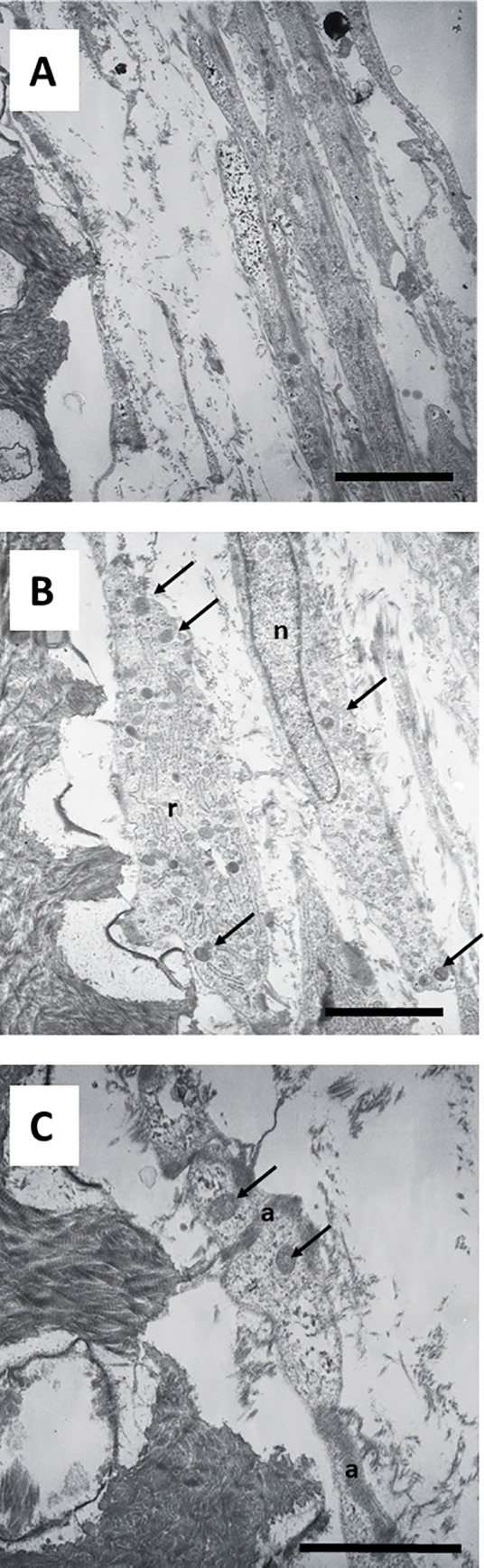
Ultrastructure of hDPSCs cultured on SB. hDPSCs were cultured on scaffolds for 6 weeks and processed for TEM. r, rough endoplasmic reticulum; a, actin filaments; g, glycogen; sv, secretory vesicles; n, nuclei. Representative results of 12 separate experiments are shown in panel A, B and C. Scale bar equals to 5 μm.

TEM analysis revealed abundant extracellular matrix deposits between flat mesenchymal-like cells with an active secretory phenotype. They were characterized by a well-developed secretory apparatus and loose chromatin nucleus. No apparent organization of collagen fibers in the extracellular matrix was observed (Fig 10A). A well-developed RER and abundant secretory vesicles were also found (Fig 10B), and abundant bundles of actin filaments were observed in the areas of intercellular anchorage (Fig 10C).

### Cellular morphology and extracellular matrix characteristics of hDPSCs grown on surface C

**Fig 11:**
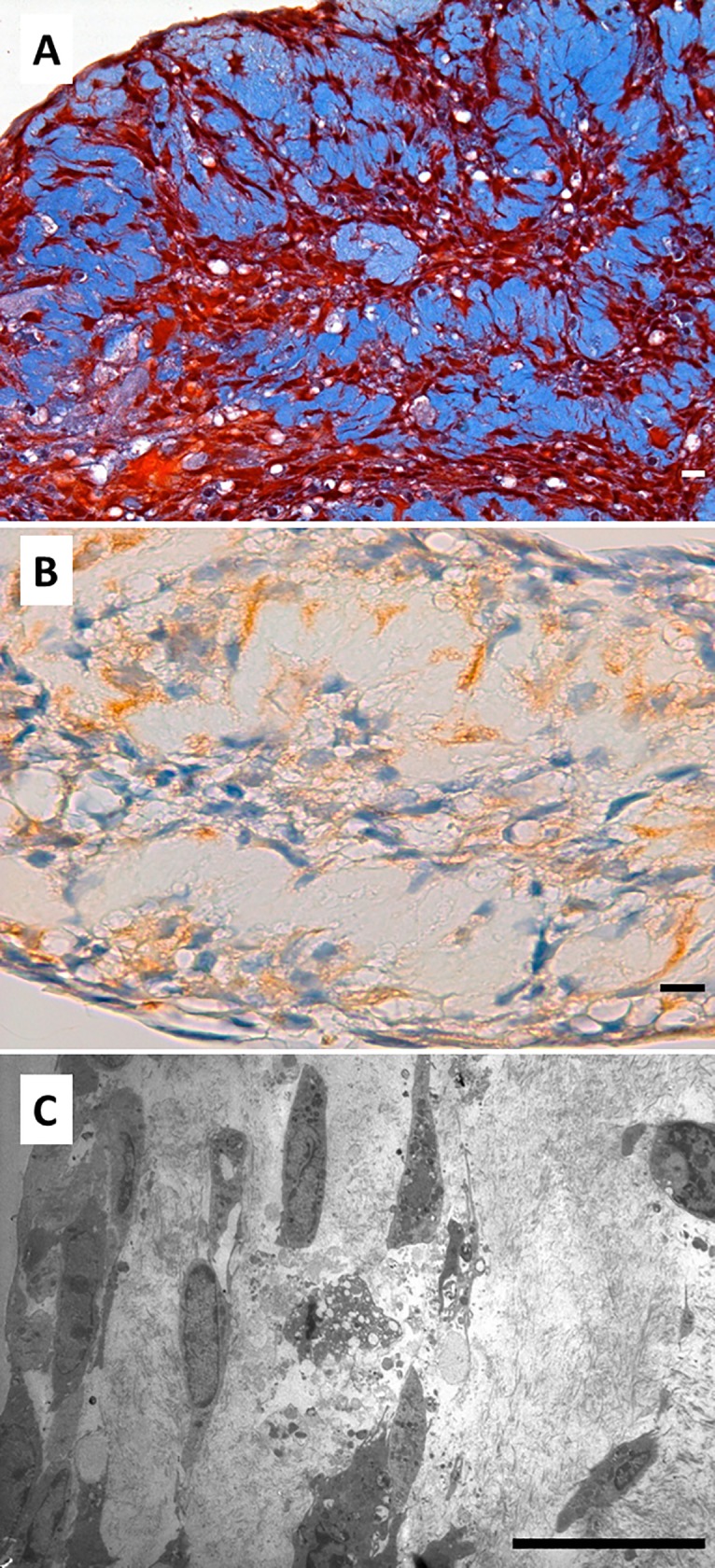
Cellular morphology and extracellular matrix characteristics of hDPSCs grown on SC. hDPSCs were cultured on the surface of the culture plate far from the dentin acellular scaffold for 6 weeks and processed for light microscopy and TEM. Samples were embedded in paraffin, cut into sections, and subjected to Masson’s trichrome staining (panel A) or immunohistochemistry for DSPP (panel B). Samples were also analyzed by TEM (panel C). Representative results of 12 separate experiments are shown. Scale bar equals to 20 μm.

Finally, our experimental model allowed us to study cells grown directly on the surface of the cell culture plates. hDPSCs grown far from the dentin scaffolds were differentiated into secretory cells, which showed an irregular phenotype and secreted an abundant matrix consisting of dense connective tissue with thick bundles of collagen fibers. They formed thick, highly cohesive cell layers. Acellular layers of connective tissue were present between them (Fig 11A). Immunohistochemical analysis revealed abundant intracellular and extracellular deposits of DSPP. The extracellular deposits were concentrated in the periphery of the extracellular matrix (Fig 11B). Electron microscopy showed the development of a characteristic secretory phenotype. These cells secreted abundant extracellular matrix generating a non-organized tissue rich in collagen fibers (Fig 11C).

## Discussion

hDPSCs are cells present in the dental pulp and show odontoblastic differentiation potential [40–41]. This property, together with their high proliferation rate and ease of isolation, make hDPSCs appropriate for dentin regeneration procedures.

A number of different strategies are available to induce hDPSCs to differentiate into odontoblast-like cells, including the use of growth factors, co-culture with other cell types, and the use of different scaffolds mimicking the dentin architecture [17, 34, 40–46]. The scaffold microstructure is a key factor in inducing the differentiation of hDPSCs into odontoblast-like cells. It is well known that scaffolds with a highly porous structure induce cell attachment, proliferation, and differentiation into odontoblast-like cell phenotypes, as reported by different groups using collagen sponges, poly-l-lactic acid, and chitosan scaffolds [33–35]. With regard to microgeometry, it seems that the presence of structures similar to dentin tubules *per se* induced the differentiation of hDPSCs *in vitro*. It has also been reported that hDPSCs grown on acellular dentin scaffolds with accessible dentin tubules develop an odontoblast-like phenotype, as characterized by light microscopy (cellular processes inside dentin tubules), DSPP expression, or alkaline phosphatase (ALP) activity [36]. However, there have been no ultrastructural studies analyzing the phenotype and secretory properties of these cells, which was the main objective of the present study. An *in vitro* odontoblast differentiation model on acellular dentin scaffolds was developed to examine this issue in depth. Our model allowed us to consider three different scenarios: non-accessible dentin tubules (SA), accessible dentin tubules (SB), and cells grown out of the scaffolds (SC). We found that the same cells cultured under the same conditions, and with the same culture medium, developed different morphologies and secreted a completely different extracellular matrix depending on the scaffold microstructure. hDPSCs grown on SA secreted a reticular extracellular matrix similar to that found in normal dental pulp tissue. Two different morphologies were observed. The cells in contact with dentin oriented perpendicular to the surface and developed cellular processes inside the dentin tubules. Both the cytoplasm and processes of these cells were positive for DSPP. These findings were consistent with data reported previously [36]. In contrast, the cells not in direct contact with dentin surfaces became stellate and most were immunohistochemically negative for DSPP. Ultrastructural analysis demonstrated a typical secretory phenotype with characteristic RER and secretory vesicles not only in the cytoplasm, but also in the cellular processes, in which it was possible to identify active secretion. Different dentin types were observed in the scaffold, including intertubular dentin and neo-dentin in the tubular walls and dentin surface, compatible with peritubular dentin. The cells studied were in intimate contact, showing the typical palisade distribution with characteristic actin accumulation in relation to sites of cell adhesion and reorganization of the cytoskeleton, as reported previously [36]. The cell processes showed a well-developed cytoskeleton and secretory vesicles, and were similar to those described in native dentin [39].

Cells grown on SB showed a completely different morphology. Layers of flat cells showing strong attachment and a high degree of organization were found, with a dense extracellular matrix rich in collagen between the cell layers. All of these cells were positive for DSPP, and large deposits of this protein were found in the extracellular matrix. TEM analysis demonstrated a characteristic secretory phenotype with well-developed RER and secretory vesicles. These cells were physically connected, and characteristic intracellular actin deposits were found in the areas of cell–cell junctions. It is important to highlight that a sharp transition between the two phenotypes was observed, as seen on analysis of slices containing both surfaces.

Finally, our experimental model allowed us to study the cells grown on SC. Interestingly, these cells differentiated into secretory cells, and expression and localization of DSPP seemed to indicate the beginning of neo-matrix mineralization. The differentiation was probably induced by growth factors secreted from cells grown on the surface of the scaffold, or by growth factors released into the culture medium from the predentin. However, further studies are needed to identify which of these possible sources was responsible for induction of the properties of these cells grown far from the dentin scaffolds.

In summary, the results presented here support the use of hDPSCs for RE and indicate the importance of both scaffold microstructure and microgeometry in odontoblast differentiation. These findings also demonstrate the development of a cell phenotype similar to native odontoblasts only on surfaces with accessible dentin tubules.

## Supporting information

S1 Fig Flow cytometric characterization of hDPSCs. Cells were isolated and cultured until 85–90% confluence. The cells were detached with Accutasse and analyzed using a cytometer equipped with a 488-nm Argon laser and a 635-nm red diode laser. Samples were gated based on their light-scattering properties in the side- and forward-scattered light modes, and 10,000 events per sample within this gate (R1) were recorded using the medium setting for the sample flow rate. Two-parameter histograms were generated according to CD45 (Pacific Blue) signal using the CellQuest software. n = 3 of each culture of hDPSCs were analyzed. Representative histograms for CD29 (Alexa Fluor 488, panel A), CD44 (PE/Cy5, panel C), CD105 (APC, panel E), CD146 (PE, panel G) and CD31 (PE/Cy7, panel I) are represented. Negative control of each of the antibodies used are also represented in panels B, D, F, H and J.
(BMP)
