# Supplementary figures and images for: Dentin tubule orientation determines odontoblastic differentiation in vitro: A morphological study
Source: PLoS One. 2019 May 9;14(5):e0215780. doi: 10.1371/journal.pone.0215780 (PMC6508697; doi:10.1371/journal.pone.0215780)

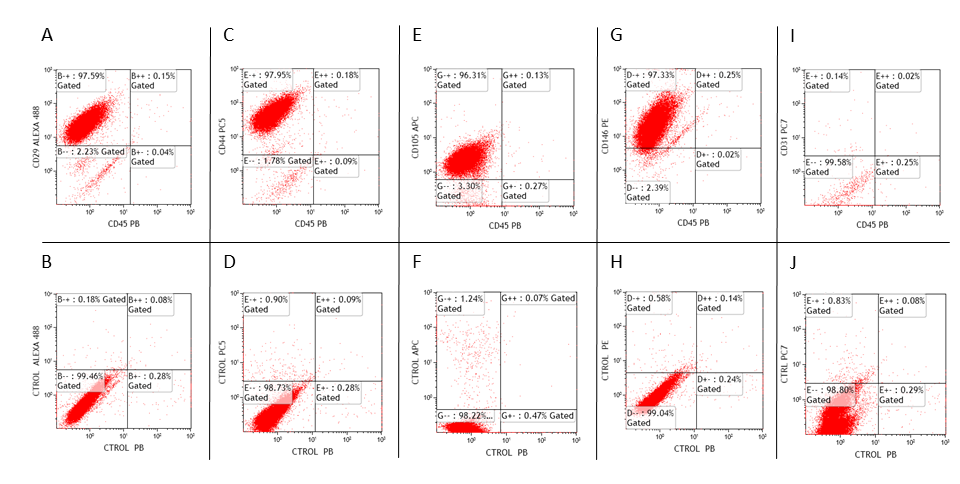

Supplement: S1 Fig — Cells were isolated and cultured until 85–90% confluence. The cells were detached with Accutasse and analyzed using a cytometer equipped with a 488-nm Argon laser and a 635-nm red diode laser. Samples were gated based on their light-scattering properties in the side- and forward-scattered light modes, and 10,000 events per sample within this gate (R1) were recorded using the medium setting for the sample flow rate. Two-parameter histograms were generated according to CD45 (Pacific Blue) signal using the CellQuest software. n = 3 of each culture of hDPSCs were analyzed. Representative histograms for CD29 (Alexa Fluor 488, panel A), CD44 (PE/Cy5, panel C), CD105 (APC, panel E), CD146 (PE, panel G) and CD31 (PE/Cy7, panel I) are represented. Negative control of each of the antibodies used are also represented in panels B, D, F, H and J. (BMP) [file pone.0215780.s001.bmp]
